# Crystals for the Microscope

**Published:** 1880-10

**Authors:** Geo. E. Blackham


### CRYSTALS FOR THE MICROSCOPE.

GEO. E. BLACKHAM, M. D., F. R. M. S.

Amidst the marvels of nature the tendency of certain classes of lifeless matter to assume certain definite shapes under given circumstances has always seemed to me among the most wonderful. This tendency, or property, is known as crystallization, and is possessed by many of the compound chemical substances known as salts, when passing from solution to the solid state and also by many, if not all, the metals when cooled from the molten condition or when precipitated in a pure state from certain of their chemical compounds. The shapes assumed by the individual crystals of any substance are regular geometrical forms, such as cubes, prisms of various kinds, &c. ; but by the combination of the individual crystals other and more complex forms in endless variety and often of great beauty are formed, as in the familiar case of the delicate tracery of the frost upon our window panes in winter.

Beautiful as many of these crystalline forms are to the naked eye they become still more so when viewed by the aid of a reasonably good microscope with a power of say, fifty, diameters, or even less; and the study of their formation and character furnishes a most attractive employment for the long winter evenings. Some hints may, perhaps, be of value to amateurs who have not as yet given attention to this subject:

Using a low power, (say the one inch objective and lowest eye piece,) prepare your microscope for observation; take a glass slide thoroughly clean and free from grease and place upon it a tiny drop of tincture of camphor and place it upon the stage. On looking through the microscope nothing will be seen at first, but in a moment the alcohol begins to evaporate and delicate feathery crystals of camphor are seen shooting across the field of view and rivaling in beauty the finest workmanship of Old Jack Frost himself; they soon fade, but the experiment is easily repeated and is worth the repetition.

A pinch of table salt in a teaspoonful of water readily dissolves, and a drop of this solution placed upon the slide and gently warmed over a lamp gives a field studded with beautiful cubes of salt of various sizes.

A tear evaporated upon a slide is a pretty object, prettier even than in the eye of beauty, though it may seem ungallant to say so.

The following list of easily obtainable salts give beautiful or curious crystals, when their solutions are evaporated upon the slide; and the forms assumed by each may be varied almost infinitely by varying the conditions under which the crystals are formed; as, for instance, the strength of the solutions, the rapidity with which they are evaporated, &c., &c.:

LIST.

Alum,
Chlorate of Potash,
Bromide of Potash,
Sal. Ammoniac,
Bichromate of Potash,
Camphor (dissolved in Alcohol,)
Common Salt,
Nitrate of Silver.

This list is simply given as a sample and might be extended almost indefinitely, but each observer will doubtless find pleasure in experimenting for him-(or her)-self.

If a drop of a strong solution of nitrate of silver be placed upon a slide and instead of evaporating it you place in it a grain or two of copper filings, the copper will begin to combine with the acid, and the silver, thus displaced from the combination, will be deposited in elegant fern-like forms of the most brilliant metallic lustre. To examine these to advantage the light should be thrown on them from above and concentrated by means of a concave mirror or a bullseye condenser. When so illuminated I know of no more brilliant sight than that afforded by the rapid growth of silver ferns which is the result of this simple experiment.

The field opened up by the study of microscopic crystallization is an extensive and attractive one, easily investigated by the beginner and ever holding out new wonders to the advanced investigator. To see the lifeless atoms in the very act of marshalling themselves in regular formations like trained battalions of intelligent beings is a marvel at which the tyro must wonder, and which the wisest can not fully explain.

